# Monitoring System for Operating Variables in Incubators in the Neonatology Service of a Highly Complex Hospital through the Internet of Things (IoT)

**DOI:** 10.3390/s23125719

**Published:** 2023-06-19

**Authors:** Pedro Antonio Aya-Parra, Andres Jacob Rodriguez-Orjuela, Viviana Rodriguez Torres, Nidia Patricia Cordoba Hernandez, Natalia Martinez Castellanos, Jefferson Sarmiento-Rojas

**Affiliations:** 1 Biomedical Engineering Program, School of Medicine and Health Sciences, Universidad del Rosario, Bogota 111711, Colombiaandresj.rodriguez@urosario.edu.co (A.J.R.-O.); 2 Biomedical Engineering Program, Engineering Faculty, Escuela Colombiana de Ingeniería Julio Garavito, Bogota 110111, Colombia; 3 Departamento de Neonatología, Hospital Universitario Mayor—Méderi, Bogota 110111, Colombia; viviana.rodriguez@mederi.com.co; 4 Departamento de Ingeniería Biomédica, Hospital Universitario Mayor—Méderi, Bogota 110111, Colombia; nidia.cordoba@mederi.com.co (N.P.C.H.);

**Keywords:** neonatal care, incubators, clinical innovation, IoMT, patient safety, medical bioinstrumentation, IoT

## Abstract

Background: Around 15 million premature babies are born annually, requiring specialized care. Incubators are vital for maintaining their body temperature, which is crucial for their well-being. Ensuring optimal conditions in incubators, including constant temperature, oxygen control, and comfort, is essential for improving the care and survival rates of these infants. Methods: To address this, an IoT-based monitoring system was developed in a hospital setting. The system comprised hardware components such as sensors and a microcontroller, along with software components including a database and a web application. The microcontroller collected data from the sensors, which was then transmitted to a broker via WiFi using the MQTT protocol. The broker validated and stored the data in the database, while the web application provided real-time access, alerts, and event recording. Results: Two certified devices were created, employing high quality components. The system was successfully implemented and tested in both the biomedical engineering laboratory and the neonatology service of the hospital. The results of the pilot test supported the concept of IoT-based technology, demonstrating satisfactory responses in temperature, humidity, and sound variables within the incubators. Conclusions: The monitoring system facilitated efficient record traceability, allowing access to data over various timeframes. It also captured event records (alerts) related to variable problems, providing information on duration, date, hour, and minutes. Overall, the system offered valuable insights and enhanced monitoring capabilities for neonatal care.

## 1. Introduction

According to the figures and data published by the World Health Organization OMS, it is estimated that approximately 15 million premature babies are born each year and it is increasing; that is, the rate of premature births is between 5% and 18% of newborns globally [1]. Approximately one million premature babies die each year due to complications in childbirth or related complications during the first days of life, with prematurity being the leading cause of death in children under five years [2].

When a newborn is born prematurely, has health problems, or is born in a difficult delivery, they are taken to the neonatal intensive care unit (NICU) (neonate means newborn) [3]. Premature babies can present certain problems in the first few days, such as difficulty keeping warm, breathing problems, and feeding difficulties. For this reason, highly specialized medical care is needed. Premature babies lack the body fat needed to maintain body temperature, and some of their organs are not fully developed to generate heat [4]. To minimize this problem, it is necessary to provide an environment in which there is the lowest possible energy expenditure, i.e., a thermo-controlled environment. For this purpose, incubators are used to provide a more suitable environment for the evolution of the babies’ health and to stabilize and maintain the thermal balance in premature babies and newborns. In 1907, Dr. Pierre Budin described the connection between mortality and temperature, finding that only 10% of babies who maintained low temperatures compared to those who maintained high temperatures survived [5].

Currently, all incubators must guarantee a series of conditions so that the baby is as comfortable as possible, isolated from noise, with optimal temperature and humidity:

- Constant temperature: Even though it is a variable temperature, it must have as few oscillations and changes as possible since it must be maintained between 28 and 34 °C.
- Oxygen concentration control: the administration of this gas to the newborn is vital to minimize the risk of respiratory failure and reduce hypoxic injuries. The saturation levels of this gas (SO2) must be monitored through a transcutaneous electrode, and the concentrations in the hood’s chamber, oxygen goggles, or endotracheal tube must be stable to avoid cyanosis, tachypnea, and apnea. A higher concentration of oxygen in the hood chamber and a lower one in the passenger compartment must be guaranteed.
- Control System: through a sensor located in the suprahepatic or lateral abdominal zone, the servocontrol allows the regulation of the baby’s body temperature.
- Visibility: it must allow good vision of the child from any angle and position to register any change in color, strange movement, or type of breathing.
- Washable and removable: It is necessary to change the incubator every seven days for a deep cleaning inaddition to its daily disinfection and the filter change as recommended by the manufacturer.
- Good access and handling of the newborn inside: The access time of the newborn must be short to reduce the loss of heat and oxygen and avoid sudden changes in temperature; likewise, the gates must allow proper handling of the baby.
- Comfortable: it must allow rest to maintain the limited energy of the newborn, isolated from the outside world, with minimum levels of noise.

In relation to the context presented, the Hospital Universitario Mayor de Mederi, regarding its newborn emergency unit, has positioned itself as a referral center in Bogotá, which brings with it an admission indicator of 18.1% of live births, both treated at the institution and referred from external units. The neonatology service is defined as the medical subspecialty derived from pediatrics dedicated to the diagnosis and treatment of pathologies in newborns during their first 28 days of life. The Neonatal Unit has indicators that show the high quality of care provided, with a neonatal mortality rate that is significantly lower than international standards of 4.3 × 1000 live births and very low rates of infection associated with health care. In relation to the installed capacity, the unit has 24 incubators whose control over operating parameters such as temperature, humidity, and sound is carried out manually at specific times by the service’s assistance personnel [6].

To improve this traceability of the incubators’ environmental conditions, a pilot test was proposed to monitor the variables available in the incubators through the Internet of Things (IoT) to provide support to specialists and take precautionary measures for safety while caring for newborn babies. The aim is to facilitate the communication of data from the neonatology area through a real-time consultation application that is accessible at any time and on a device with Internet access, generating a complementary strategy to the process of continuous improvement of the neonatal service and the Mederi hospital under the need for interoperability.

In this study, an Internet of Things (IoT)-based monitoring system was developed and implemented to monitor sound, temperature, and humidity variables in a hospital environment. Extensive tests were performed to assess the accuracy and correlation of the captured data with the programmed values in the incubators. A web application was designed and developed to allow users to register and access the platform with authorized credentials. The application provided graphical reports and statistics so support staff and engineers could make timely decisions. The IoT-based monitoring system design was carried out in a university hospital environment, allowing biomedical engineers and health personnel to access incubator-related information in real time.

## 2. Previous Works and Literature Review

In recent years, the healthcare industry has undergone significant changes, driven to a large extent by technological advances that have transformed the traditional hospital-centric approach into a patient-centric approach. The use of small-sized devices and remote health monitoring have allowed the diagnosis of diseases, health control, and the reduction of health care costs [7]. These advances are especially relevant in studies about population aging and the growing prevalence of chronic diseases. In this sense, remote health monitoring, by collecting physiological data and providing real-time feedback to healthcare personnel, is presented as a promising solution to improve patient care and minimize hospital visits. This approach has the potential to relieve pressure on healthcare facilities, especially in critical services, rural and remote areas, and improve the quality of life for patients by offering more personalized and accessible care.

Health risk management is a complex challenge that requires the development of effective systems to ensure safety in each clinical setting. Thus, it is crucial to identify critical processes, analyze them, and find tools and solutions that reduce risk, simplify management, and ensure traceability of activities related to monitoring at the hospital level. The advantages of technology, especially the Internet of Things (IoT), allow advances in the use of wireless sensor networks (WSN) and the availability of integrated devices, cloud services, sensors, actuators, and other devices of low cost and power, providing the opportunity to develop smart solutions that improve risk-oriented management to ensure quality in the provision of health services.

As stated in previous research [8], Cold Chain Management stands out as a relevant issue and can be addressed in the proposed work. Hence, the implementation of a real-time monitoring system for drugs, vaccines, medical devices, and biological samples (blood and its derivatives, saliva, urine, and cells of various types and classifications). The proposed system offers immediate alarm tools in the event of a malfunction of refrigeration systems (refrigerators and/or freezers).

According to Almaiah, the advancement of fifth-generation communication networks and the upcoming arrival of sixth-generation networks have fueled the concept and use of the Internet of Things (IoT), leading to great potential to revolutionize healthcare as we know it. One example of this is telemedicine, powered by IoT technology. The use of these technologies has become significant in medical applications that somehow bridge the gap between rural and urban areas in terms of access to healthcare. Telemedicine offers advantages such as low-cost consultations, remote examinations, and diagnoses, allowing leading experts and physicians to reach remote areas and provide advanced services at an affordable cost. The combination of IoT and real-time computing solutions plays a crucial role in the health sector, and advanced computing technologies have been proposed to promote smart health. These advances have the potential to improve the accessibility and quality of medical care, providing significant benefits at both the care and population levels [9].

In an exploratory review carried out in [10], sensors play a fundamental role in the diagnosis, treatment, and long-term follow-up of diseases, as well as in the observation and evaluation of the physiological activities of patients in critical conditions. Together with the Internet of Things (IoT) and artificial intelligence (AI), sensors have become the core of next-generation healthcare technologies, from which clinics and hospitals will be the main beneficiaries. Under the study context, biocompatible biosensors have emerged as a promising solution to monitor long-term physiological information (medical services), providing advantages in terms of accurate detection and reliable acquisition of the physiological behavior of the human body. These biosensors are used to measure vital parameters, biochemical indicators, and other physical and physiological parameters relevant to medical care. Its development, without a doubt, revolutionizes the medical care system by providing more advanced and accurate monitoring. However, they also face challenges and opportunities in their future development. Overall, biocompatible sensors represent a promising technology that has the potential to significantly improve healthcare and improve disease diagnosis, treatment, and monitoring.

The high population in different countries worldwide has led to different problems at the social level, such as health care, food security, and schooling, among many other aspects. As a result, previous researchers [11] have presented an alternative in terms of monitoring physiological variables, where the objective is to read and analyze the vital signs of patients and reduce latency during signal transmission. The above had as an initiative real time help in personal care based on the internet of things technology.

According to previous research [12], heart rate is a fundamental parameter to know the health state and the main cardiovascular risks, especially when it can be continuously monitored using portable devices in daily life. In this way, several investigations use photoplethysmography (PPG) through smart devices, such as smartwatches, for continuous monitoring of respiration; this is how they are based on a certain medical discovery called respiratory sinus arrhythmia (RSA), which describes the relationship between respiratory and heart rate.

Based on the above, the research team developed BreathAnalyzer, a smart watch that integrates an artificial learning model based on decision trees that adapts to the characteristics of multiple domains while considering the limitations of the smart watch. Based on the above, it can be understood that the BreathAnalyzer prototype embedded in the smartwatch, in conjunction with a thorough evaluation, demonstrates that the BreathAnalyzer outperforms state-of-the-art approaches, with an accuracy improvement of 35.37% to 80%. An average of 42% was obtained in a variety of practical scenarios, such as identifying potential arrhythmias.

Based on the great needs that currently exist in the health sector, different health care experts are developing different smart devices and other medical technologies, as well as ambient intelligence, a key factor in the use of technologies such as the Internet of Things (IoT) [13]. Remote health surveillance can be used to track non-critically ill patients as opposed to referring them to a hospital, relieving pressure on hospital services, optimizing resources, and lowering the burden on healthcare professionals. In this way, the use of information and communication technologies can be used to improve rural residents’ access to healthcare or to allow older people to remain monitored at home for longer periods of time. Essentially, it can increase access to clinical services, thus relieving pressure on health facilities, and it can empower people to always have more autonomy over their own well-being.

Another technological advance of recent years, as presented in [14], analyzes the design and implementation of a telemedicine health monitoring system (THMS) based on the Internet of Things (IoT) with an early warning scoring (EWS) function that reads, evaluates, and records a patient’s physiological parameters, such as body temperature, oxygen saturation level, systemic blood pressure, breathing patterns, pulse (heart) rate, dependency of supplemental oxygen, consciousness, and pain level, using particle photon microcontrollers interfaced with biosensors and switches.

Based on paired sample t-tests obtained from six sessions with 10 trials for each vital sign per session, there were no significant differences between the clinical data obtained from the prototype and commercially sold medical equipment. The mean differences between the samples compared for each physiological data were not greater than 0.40, the standard deviations were less than 2.3, and the p-values were greater than 0.05. With an accuracy of 96.67%, the FI-MDSS predicted levels of health risk that were comparable to conventional EWS techniques.

## 3. Materials and Methods

This study focused on the development and implementation of a monitoring system based on the Internet of Things (IoT) to monitor sound, temperature, and humidity variables in a hospital environment. Extensive tests were performed to assess the accuracy and correlation of the captured data with the programmed values in the incubators. A web application was designed and developed that allowed users to register and access the platform with authorized credentials. The application provided graphical reports and statistics so that support and engineering staff could make timely decisions. The design of the IoT-based monitoring system was carried out in a hospital-university environment, where it allowed biomedical engineers and healthcare personnel to access information related to incubators in real time.

The importance of monitoring the temperature, humidity, and noise in the incubators of the neonatology service is based on the need to provide an optimal and safe environment for premature newborns. These variables are critical to the well-being and health of babies in the neonatal intensive care unit.

Temperature is a key factor in the development and survival of premature babies. Maintaining an adequate body temperature is crucial to preventing hypothermia or hyperthermia since premature babies have a limited ability to regulate their body temperature. Studies have shown that hypothermia and hyperthermia can increase morbidity and mortality in preterm infants [15]. The relative humidity of the air also plays a crucial role in the care of newborns in incubators. Premature babies have thinner, more fragile skin, making them more susceptible to water loss and dehydration. Maintaining an adequate level of humidity in the environment helps prevent excessive moisture loss through the skin and reduces the risk of dryness and damage to the respiratory tract [16]. Excessive noise in the neonatal intensive care unit environment can have adverse effects on premature babies. Studies have shown that exposure to high noise levels can cause physiological stress, sleep disruption, and impaired hearing development in newborns [17].

**Figure 1:**
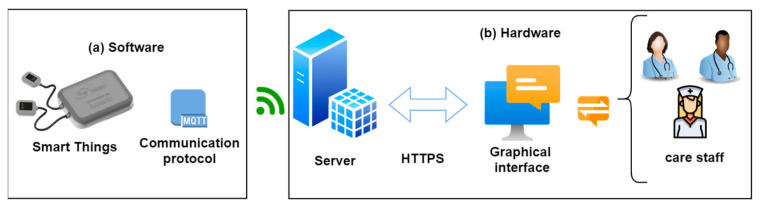
Design of the IoT system connected to healthcare personnel: (**a**) hardware components and (**b**) software components.

As shown in Figure 1, the system includes two main components, both hardware and software, which were developed with open-source technologies contemplating the user experience, scalability, and robustness under the current standards of IoT system development. This will allow for the future development and implementation of more Smart Medical Devices and technologies under standardized protocols and with a platform that can be scaled to other services.

### 3.1. Hardware Development

**Figure 2:**
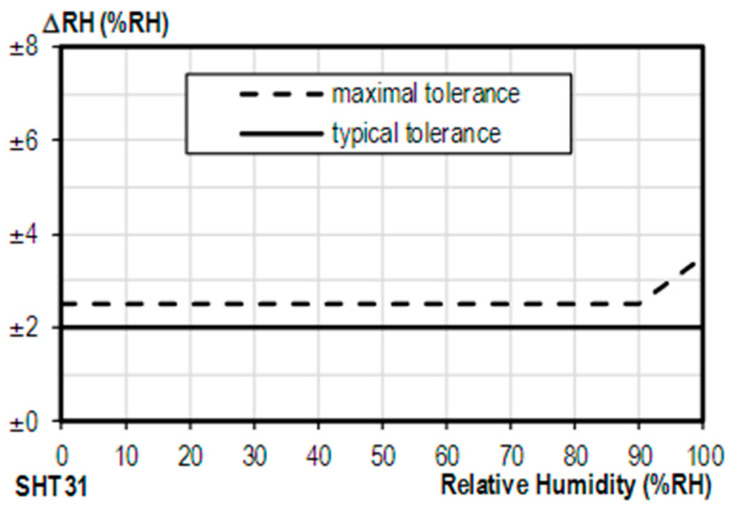
Accuracy and tolerance to humiditySHT31 [19].

Regarding the materials used for the construction of the electronic device, a microcontroller with reduced dimensions and high processing characteristics was used. In this way, the electronic components used were selected according to the needs of the project. Next, in Table 1, the main characteristics of each of the components are described:

The main component used to perform the measurements is the SHT31 temperature and humidity sensor. This has an accuracy of 1.5% RH for the relative humidity measurement, its standard output curve is presented in Figure 2 with its allowed tolerance.

**Figure 3:**
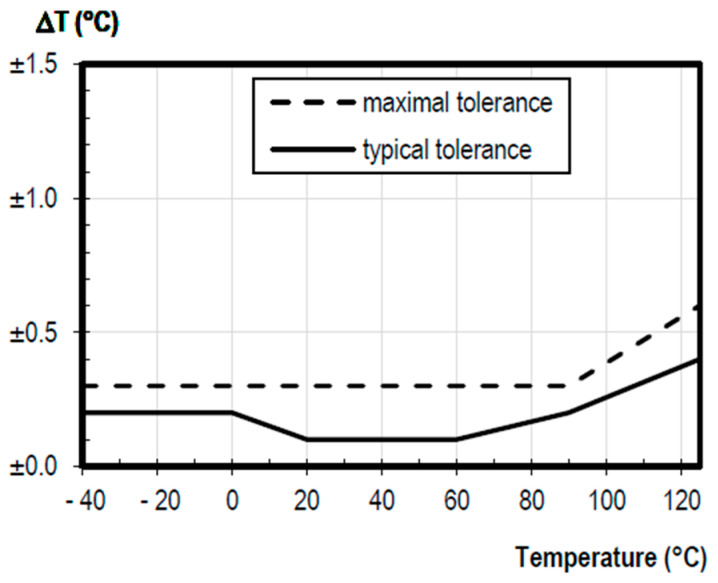
Accuracy and tolerance to temperature [19].

On the other hand, for the temperature measurement, the sensor presents a resolution of 0.2 °C, its standard output curve is presented in Figure 3 together with its tolerance. This means that the sensor can detect very small changes in ambient humidity and temperature.

**Figure 4:**
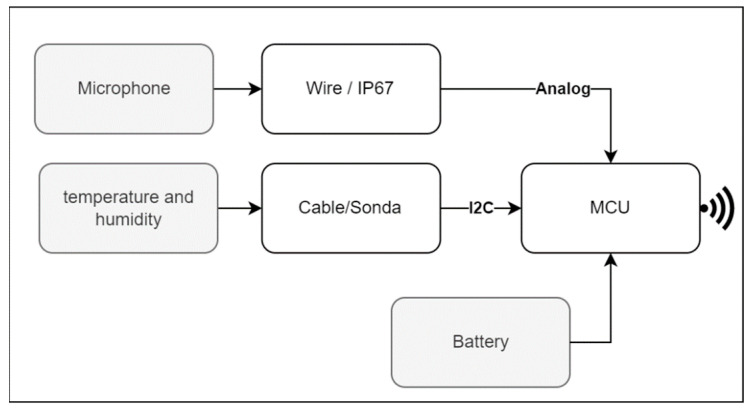
Logging system hardware description.

The device that captures the information consists of four components: two sensors, a microcontroller, and a battery. The temperature, humidity, and sound sensors were connected to the microcontroller (MCU), which is characterized by having an easy, secure, and reliable connection to the internet via WiFi [23]. The interconnection process between the system components is described in Figure 4.

Once the device is turned on, the microcontroller establishes a connection to the previously configured WiFi network. The values of the variables of sound, temperature, and humidity are obtained, converting the voltage signals to digital format for their subsequent transmission through the Internet in fragments or data packets in JSON type format. This operation is performed at a frequency of 5 min.

The information about the set of variables is published using the MQTT communication protocol to a broker EMQX [24], which is implemented on a server with public access [25,26]. Next, the three parts of the system are described, and their configuration and performance are shown in general.

**Figure 5:**
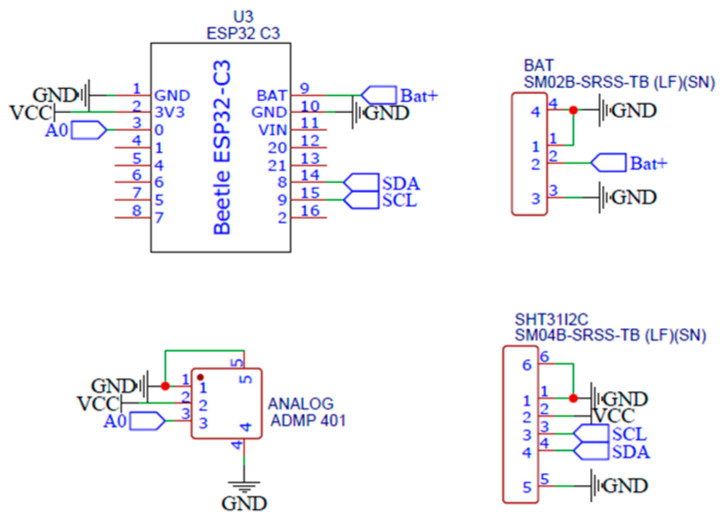
Schematic of the developed device.

As can be seen in Figure 5, each of the sensors was connected to the microcontroller using both analog and digital communication protocols, such as I2C. This connection was made physically using high quality probes and their corresponding input and output connectors. These cables were characterized by their ability to transmit information with excellent quality and their reliability due to their conductivity and durability properties.

**Figure 6:**
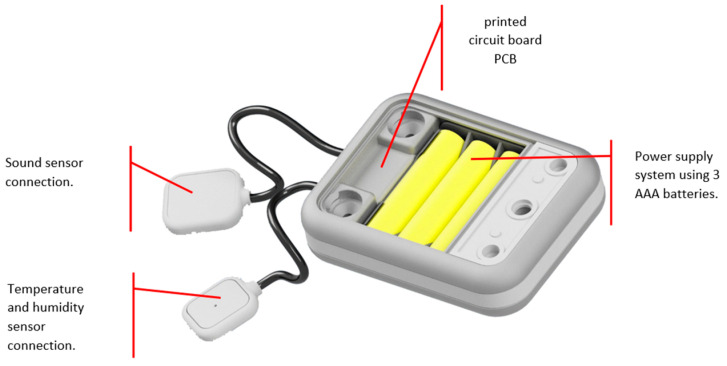
Internal description of the device.

Regarding the power supply, three conventional batteries were used that offered a performance of approximately 15 days. Figure 6 shows the functional device, which is protected by a custom-made casing or box made using 3D printing with PLA material.

**Figure 7:**
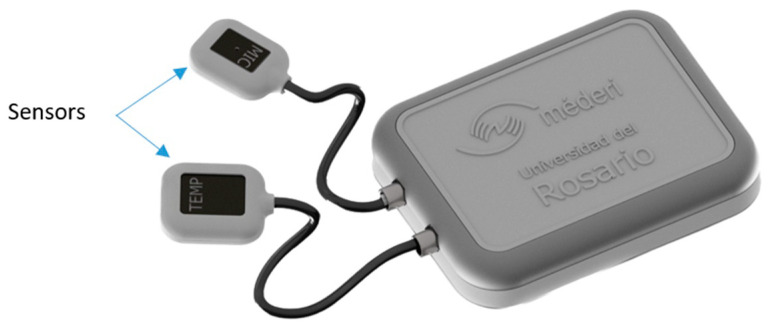
A general device with its sensors.

Figure 7 shows the final design with its protective cover, with the main objective of keeping each of the electronic and transit components in position for easy cleaning and disinfection since it is a device that was used in a hospital environment. In fact, for each of the sensors, its protective box was designed and printed with the purpose of protecting its electronic parts and, in some way, guaranteeing their proper functioning and durability.

The device has reduced dimensions of around 12 cm long × 8 cm wide, and 2 cm thick. The ideal of the device is that it be light, small, and compact. Placement of the fastening system in the incubator was carried out by means of two silicone suction cups located on the back of the device.

### 3.2. Software Development (Backend)

**Figure 8:**
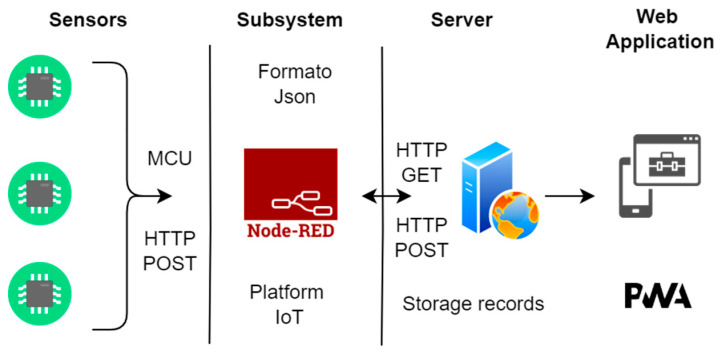
Network broker node operation.

The function of the broker was to guarantee the quality of the data acquired before its publication on the interface. This process made it possible to identify possible errors in the reading of the sensors or the loss of data. If losses were detected, they were debugged and corrected for later display on the interface. This type of architecture, presented in Figure 8, is known as centralized.

Once the information was validated by the broker, it oversaw the sending of the respective records to the previously configured database on the server. In this way, the values of temperature, humidity, and sound [27] were sent and received using the MQTT communication protocol. Once the data was received in the cloud, the processes began to generate a multiplatform interface that allowed observing the behavior of temperature, humidity, and sound, and generating alerts through notification messages on the interface for healthcare personnel.

### 3.3. Web Application Description (Frontend)

**Figure 9:**
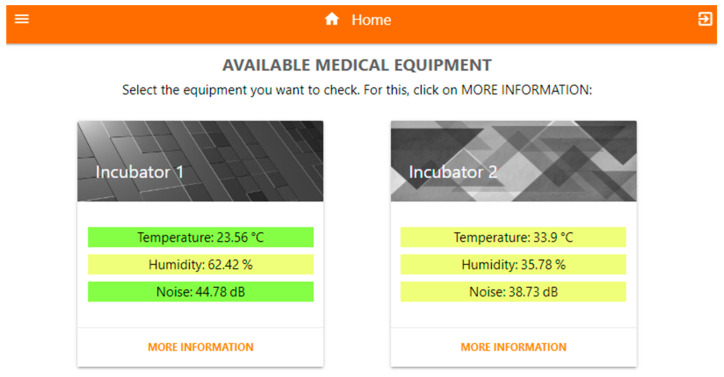
SiMCa-Bio monitoring system.

A progressive web application (PWA) was developed (SiMCa-Bio) to allow the visualization of the different monitored incubators and their measurements in real time by the healthcare and engineering staff of the hospital. This application integrates different markup languages, such as HTML and CSS, and programming languages, such as Node.js, PHP, and JavaScript, as shown in Figure 9.

To guarantee the secure storage of the data in the system, a relational database was implemented using MySQL [28], which is hosted on an external server. To store the information in the database, the broker communicates and transfers the data through the HTTP protocol using an SSL security certificate. This communication is conducted through an API created in PHP, a server-side programming language, in charge of storing the information in the relational database.

**Figure 10:**
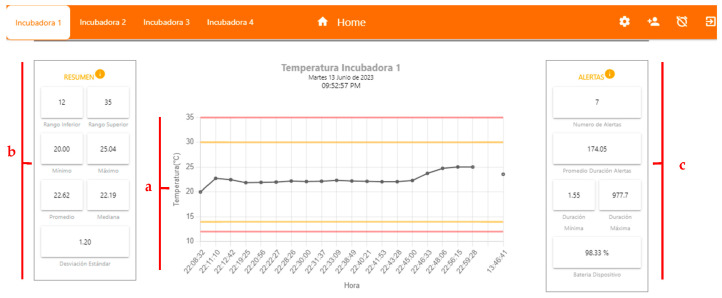
User interface with relevant indicators. (**a**) Data consulted in real time and presented interactively in a control panel; (**b**) Current statistics by date; (**c**) Alerts.

The main objective of the server side was to acquire the data from the variables, store it in an SQL database, and create a PWA interface from which it could be consulted in real time and presented interactively in a control panel (Figure 10a). This interface displayed current statistics by date (Figure 10b), such as maximum, minimum, average, standard deviation, date, and time values, among other relevant data.

In addition, the recording and presentation of the alerts generated when the programmed values in the incubator were outside the established ranges were considered (Figure 10c). These records included information such as the type of variable, the reported value, the duration of the alert, and other relevant data. Every time an alert occurred in any of the variables, a notification was automatically sent to the care staff so that they could take quick action and perform a review of the equipment.

## 4. Results

**Figure 11:**
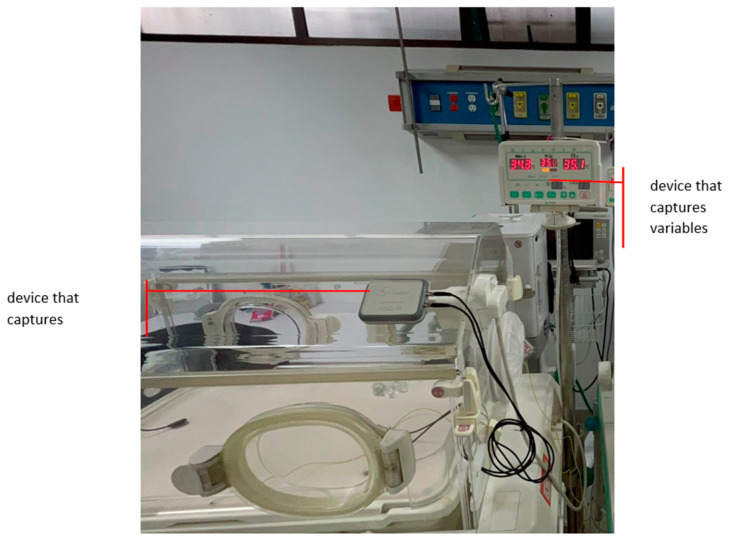
Location of devices in the incubator.

The device was programmed to perform a sampling of the variables every 5 min, during the incubator’s operating tests after having carried out the corresponding preventive maintenance. These tests lasted approximately 4 h where data was taken manually, that is, in parallel while the monitoring system automatically recorded the information, to perform a correlation test of the data from the monitoring system and what was reported (scheduled) in the incubator, as presented in Figure 11.

**Figure 12:**
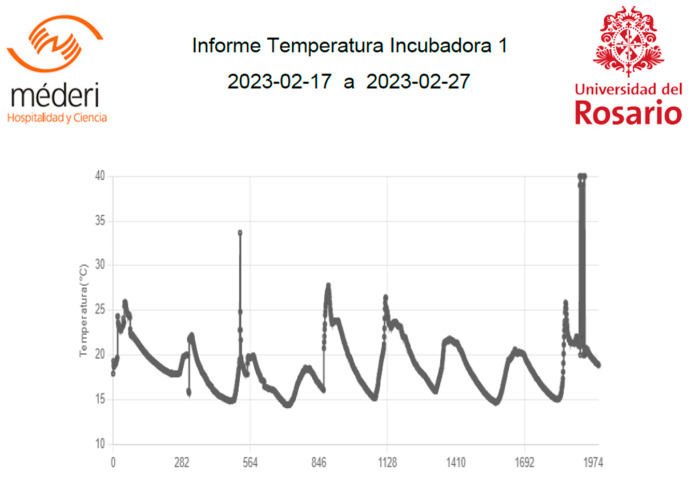
Behavior of the temperature variable.

**Figure 13:**
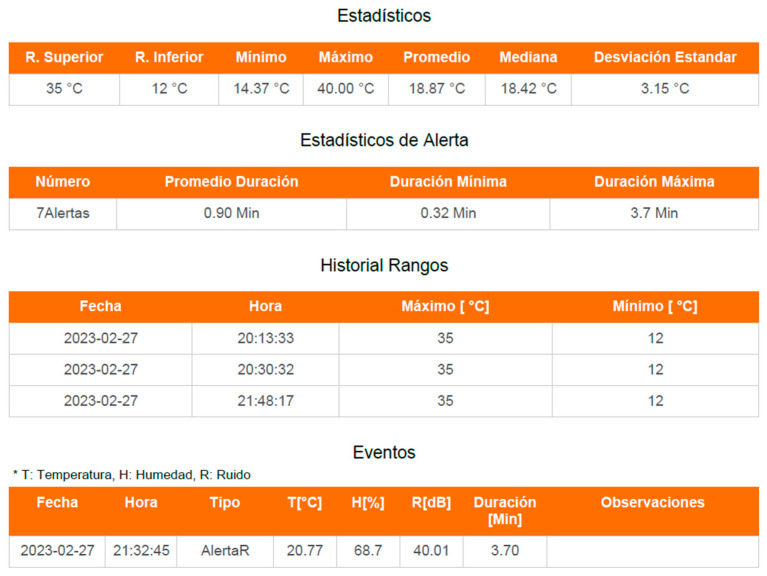
History of generated alarms.

A continuous test was carried out on the sending of data, alert generation tests, and battery charge and discharge system tests. As a result, in Figure 12 and Figure 13, the report that was generated directly from the web application is presented, where the tests taken on the variables of temperature, humidity, and noise can be evidenced, as well as the established parameters, as well as the generation of alerts and duration of these.

### Information Collection and Systematization Procedure: Monitoring Period

**Figure 14:**
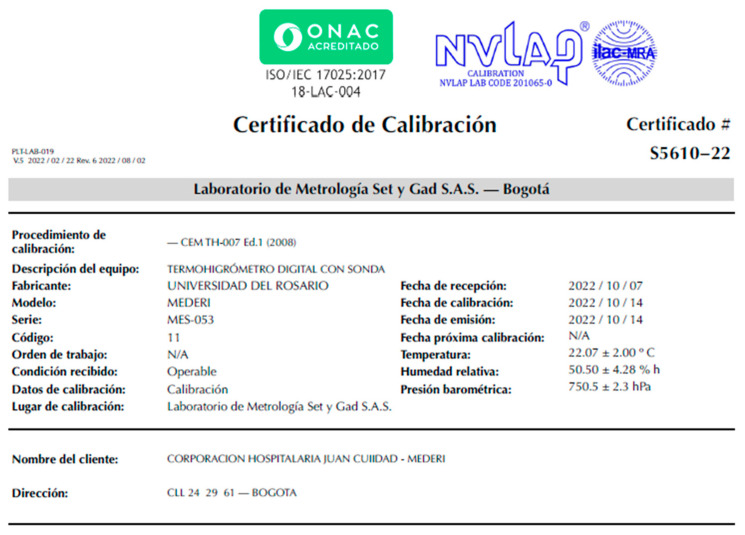
Calibration certificate for the temperature and humidity sensors.

The functional tests of the system were carried out for four months during which the tests of continuous sending of data, generation of alerts, and charge and discharge of the battery were carried out. This was conducted in the maintenance laboratory of the hospital’s biomedical engineering department in a controlled and supervised environment. During these tests, the devices captured information on the variables of sound, temperature, and humidity autonomously, and records were taken manually (on paper) in relation to the variables that the incubator had since not all the equipment had sound recording. In this way, the device was exposed to a calibration test under the ISO/IEC 1725:2017 standard to guarantee its measurements (Figure 14).

The second stage was developed in the neonatology service of the Mederi major university hospital, under the supervision of the head of the service and healthcare personnel, where the devices were in Atom brand incubators, which were not in use but were located within the service. The intention was to use the equipment in a real environment without putting the life of a newborn at risk.

**Figure 15:**
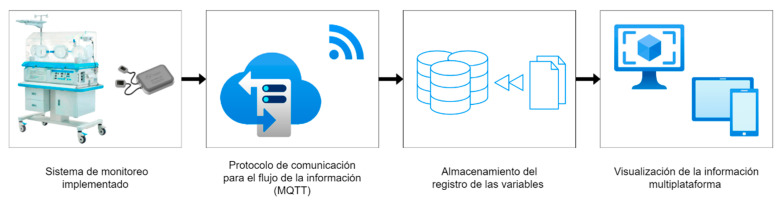
Flow of information captured from the device to the cloud.

Once the monitoring system was in the incubator, it was turned on. The system is configured to automatically connect to the nearest WiFi wireless network (Mederi). From the above, the system began to record the data on the operation of the incubator at a sampling frequency of every 5 min; this information was stored directly in a database that was hosted in the cloud (Universidad del Rosario Server with restricted access according to institutional policies). Figure 15 shows the general system, where the data that the system recorded and later consulted were: date, time, temperature, relative humidity, and sound.

The data was acquired from the incubator with a sampling frequency of 5 min, after which the device automatically connects to the available WiFi network of Mederi, and through the MQTT protocol, it sends the collected data to the database of the cloud of the Universidad del Rosario. Through the structuring of JSON-type files, the consultation of these data was carried out in real time through a responsive web interface (adaptable to different screen sizes) to the database, where the behavior of each of the variables is observable. On the other hand, it allowed the configuration of alarms, alert histories, and alerts in real time for a device with abnormal variables. An abnormality in the equipment is understood as any data that is outside the ranges defined by the institution or Colombian regulations.

The consultation of the database is only allowed by administrators of the application. The visualization, consultation, and reception of alerts will be handled by the authorized assistance personnel and the engineering personnel of the hospital designated for the pilot study.

**Figure 16:**
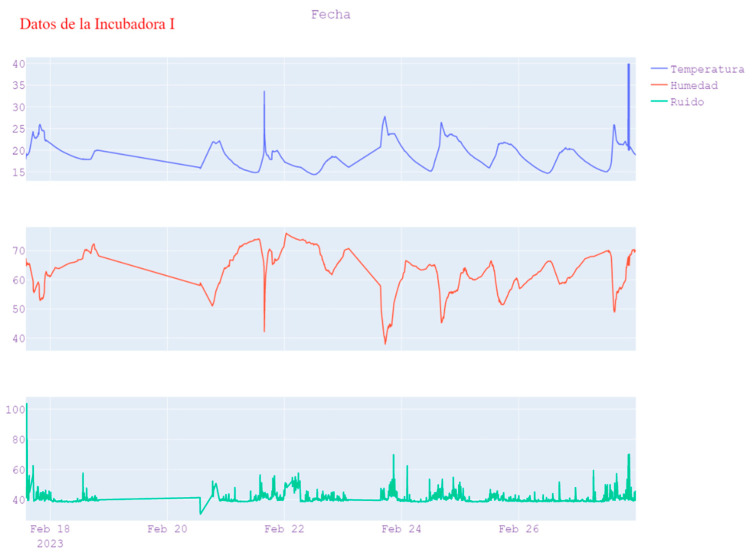
Variable behavior.

To analyze the data, the trends in each of the measurements were observed over time (the sending rate was programmed with a frequency of 10 min sending each data). Figure 16 shows the plot of temperature, humidity, and noise level as a function of time; a total of 1989 samples registered by the developed system were obtained, this allowed for the identification of any patterns or correlations between the different variables. Similarly, summary statistics such as the mean, median, and standard deviation of each variable were calculated to obtain an idea of the overall distribution of the data.

The description of the measurements obtained can be seen in the following Table 2:

Two tests were carried out with two “Atom Brand Incubators” according to the standard operating protocol used by the group of engineers to carry out functional tests. The tests were carried out under the supervision of the engineers of the area where the developed system was tested in a real environment. Tests were carried out on March 16, where a total of 25 samples were obtained during manual registration and a total of 94 samples were registered by the developed system, for which a grouping was carried out by specific hours and minutes with manual records, as seen in Table 3.

**Figure 17:**
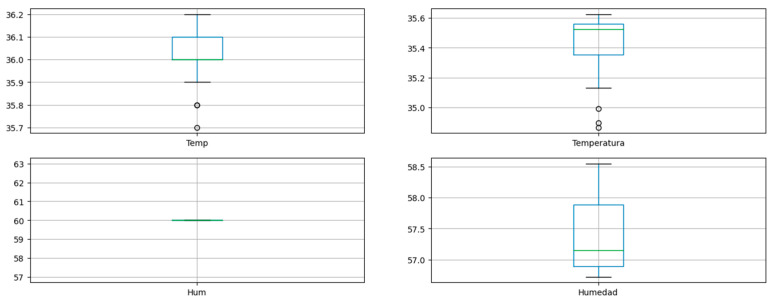
Normality analysis on test data.

A paired means t-test was used for the analysis of normality and, with this, the subsequent calculation of the correlation. Figure 17 shows the distribution of the data below:

Where temp and hum correspond to data collected manually. In the case of temperature and humidity, a comparison of means was performed through a t-test for related samples in Table 4 and in contrast to the graph, the normal distribution of the data can be verified.

As can be seen in the graph and in the previous table, all the measurements present a normal distribution, so the Pearson correlation coefficient is calculated in Table 5.

Therefore, a strong correlation can be established between the two temperatures with a significant *p* value. In the case of humidity, since the manual recording does not vary during the experiment, the correlation coefficient could not be calculated; therefore, a mean check of the measured values was carried out with the following equations.

$Absoluteerror\left(\epsilon_{a}\right):$

(1) $$\epsilon_{a}=\frac{1}{n}\sum_{i=1}^{n}{\mathrm{HumidityManual}}_{i}-\frac{1}{n}\sum_{i=1}^{n}{\mathrm{HumiditySensor}}_{i}$$

where:

n: number of data samples

$\epsilon_{a}=\;60\;\%\;-\;57.48\;$

$\epsilon_{a}=\;2.52\%$

 $RelativeError\left(\epsilon_{r}\right):$

(2) $$\epsilon_{r}=\;\frac{\epsilon_{a}}{\frac{1}{n}\sum_{i=1}^{n}{\mathrm{HumidityManual}}_{i}}$$

$$\epsilon_{r}=\;\frac{2.52\;}{60}=4.2\%$$

From this result, it is understood that it is within the range established at the beginning of the protocol.

In the future scenario of continuously implementing the system developed in the neonatal service, it can be inferred that there will be a difference in the correlation of the variables. This suggests that the specific characteristics of the environment and the use dynamics of the equipment in the service (incubator) affect the value of the variables measured in the incubators and the device. However, to reach a meaningful conclusion on this premise, a clinical study is necessary.

By analyzing the measurements of temperature, humidity, and noise level at different times, patterns and correlations between these variables were identified. To better understand the distribution of the data, descriptive statistics for each of the variables were calculated.

## 5. Discussion

The development of a new electronic device leads to different challenges, especially if it is for clinical use. Currently, there are companies that provide monitoring services for environmental variables such as temperature and humidity. However, the challenge at the clinical level entails some important considerations, especially when it comes to guaranteeing the ideal conditions that a biomedical team must meet to guarantee the well-being of a patient. Under this scenario, the monitoring system developed has both a technical and clinical scope since it is about establishing a technology-based tool that helps engineers and healthcare personnel have greater control over the operation of their equipment, as indicated by Pise, A., in his work.

It should be considered that currently there are no devices or systems on the market that properly adjust to the needs of the medical field, which translates into an imminent need to investigate and work on new developments that allow to support or solve the different problems in the hospital ecosystem.

From this perspective, the use of robust and efficient technologies is a fundamental part of the different efforts to develop real-time monitoring systems that allow decision-making in an agile manner. In other words, the use of the Internet of Things in the medical field (IoMT) is key when it comes to generating technological proposals that help implement comprehensive solutions. In relation to this technology, the Internet of Things was used in the present project as a source of communication between the information captured in each of the incubators and the final supervision through the user interface generated in the web application.

In this sense, it was possible to have a more direct approach between the developed monitoring system and both clinical and engineering personnel, allowing them to consider the multiple utilities and benefits that the technology applied to the institution’s biomedical devices and equipment can provide, which translates directly into an environment of interoperability between medical teams and care services.

Based on what has been described, the tests carried out over a short period of time generated excellent results in terms of transfer, debugging, and storage of data according to the captured variables. Consequently, the reported records of temperature and humidity were correlated with those programmed and registered on the screen of the incubators, which allowed corroborating that the data were very similar (low error), and on the other hand, the execution of the alert system was put to the test once an abnormal value was registered in the incubator’s operation.

Finally, regarding this aspect of alerts, it is possible to configure different scenarios from the web application of the monitoring system, such as incubator maintenance, user management, alert configuration, among others.

Compared to the mentioned background, the current project focuses on the development and implementation of an IoT-based monitoring system for sound, temperature, and humidity variables in a hospital environment. The need to adjust existing devices and systems to the needs of the medical field and the importance of developing integral solutions to guarantee the proper functioning of the equipment and the safety of the patients are highlighted. In addition, the use of a web application is mentioned so that assistance personnel can access and use the monitoring system, as well as generate graphic reports and statistics to facilitate decision-making. The current project builds on previous work that has explored the use of IoT technology in monitoring physiological and health variables. However, it differs by focusing on the hospital context and on the monitoring of specific variables such as sound, temperature, and humidity.

## 6. Conclusions

As a result, it can be affirmed that it was possible to develop and implement a system under the Internet of Things technology that allows monitoring the variables of sound, temperature, and humidity, in this case, in a hospital environment. Based on the above, it was possible to guarantee the proper functioning and traceability of the variables in question, according to the tests carried out.

Besides, it was possible to design and develop a web application where users (assistance personnel) must register through the web application (credentials) in such a way that the system administrator (a designated official in the service) enables the entry of each user into the application. Likewise, the application provides a graphic report and general statistics of the behavior of each variable, which allows healthcare and engineering personnel to make decisions in a timely manner to guarantee the proper functioning of the equipment and patient safety.

To conclude, the monitoring system allows efficient traceability of each of the records taken by the device in such a way that the records can be consulted from a short period of time, such as one day, to longer periods of time, such as a month or multiple years. Further, it was also possible to obtain the records of each of the events presented (alerts) generated by the device that account for the different problems presented in each of the variables, with their duration, date, hour, and minutes.

## Figures and Tables

**Table 1:** Hardware components.

Sensors Box	Component	Specifications
**ESP32-C3**	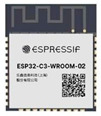	The E card SP32-C3 is ideal for IoT applications with support for dual-band WiFi and Bluetooth 5 (LE). This improves connectivity and allows for more stable communication, even in longer-range areas. In addition, it supports Bluetooth Mesh and Espressif WiFi Mesh protocols, making it easy to create networks and extend coverage [18].
**SHT31 Temperature/Humidity Sensor**	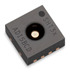	The SHT31 sensor is a highly integrated solution that combines capacitive components for humidity sensing and bandgap temperature sensing components. It supports I2C communication and can be used with 3.3V/5 and ESP32 controllers. This sensor provides high accuracy in humidity measurement, with a margin of error of ±2% in a range of 0% to 100% (at 25 °C) and a typical accuracy of ±0.2 °C in a 0 °C to 90 °C temperature range [19].
**Sound sensor** **ADMP 401**	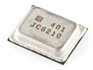	This board features an OpAmp to bring the microphone output down to a usable level, allowing it to be connected directly to an ADC on a microcontroller. In addition to amplifying the signal, the OpAmp adds a bias voltage of 5 VDC, which means it picks up sound sources from all directions [20].
**Two-pin retention button**	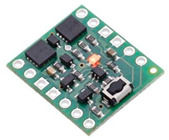	Two-position plastic button for turning on and off systems with low current consumption [21].
**Cable Assemblies** **Circular Cable Assemblies**	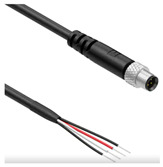	Cable/Connector, 4 Wire M5 Type Plug, 5 × L14.8 mm, Silver Plated, Molded Style, Solder Cup, IP67 [22].

**Table 2:** Record of characteristics measured by the device.

Variables	Device MAC	Temperature	Humidity	Noise	Battery
count	34:B4:72:01:CC:C0	1989	1989	1989	1989
mean	34:B4:72:01:CC:C0	19	64	41	3.3
std	34:B4:72:01:CC:C0	3	7	5	3.3
min	34:B4:72:01:CC:C0	14	38	30	3.3
25%	34:B4:72:01:CC:C0	16	60	39	3.3
50%	34:B4:72:01:CC:C0	18	64	40	3.3
75%	34:B4:72:01:CC:C0	20	69	41	3.3
max	34:B4:72:01:CC:C0	40	76	104	3.3

**Table 3:** Monitoring system record table.

Hour	Temperature Sensor	Manual Temperature	Dif (TS-TM)	Humidity SENSOR Sensor	Manual Humidity	Dif (HS-HM)
12:40	34.87	35.70	−0.84	58.51	60.00	−1.49
12:50	34.90	36.00	−1.10	58.55	60.00	−1.45
13:00	35.13	36.20	−1.07	58.43	60.00	−1.58
13:10	35.27	36.00	−0.73	58.38	60.00	−1.62
13:20	35.33	36.00	−0.67	58.16	60.00	−1.84
13:30	35.38	36.00	−0.62	57.99	60.00	−2.02
13:40	35.48	36.10	−0.62	57.78	60.00	−2.22
13:50	35.48	36.00	−0.52	57.61	60.00	−2.39
14:00	35.53	36.10	−0.58	57.61	60.00	−2.39
14:10	35.55	36.00	−0.45	57.40	60.00	−2.60
14:20	35.54	36.00	−0.47	57.26	60.00	−2.74
14:30	35.52	36.10	−0.58	57.15	60.00	−2.85
14:40	35.53	36.00	−0.48	57.09	60.00	−2.91
14:50	35.56	35.90	−0.34	57.05	60.00	−2.95
15:00	35.56	36.20	−0.64	56.99	60.00	−3.01
15:10	35.57	35.80	−0.23	56.91	60.00	−3.09
15:20	35.56	36.10	−0.54	56.91	60.00	−3.09
15:30	35.63	36.10	−0.48	56.87	60.00	−3.13
15:40	35.59	36.00	−0.41	56.84	60.00	−3.16
15:50	35.61	36.10	−0.50	56.73	60.00	−3.27
16:00	35.56	36.00	−0.44	56.72	60.00	−3.28
16:10	35.57	36.10	−0.53	56.76	60.00	−3.24

**Table 4:** Normality test.

	Kolmogorov-Smirnov ^a^			Shapiro-Wilk		
	Statistic	1gl	Sig.	Statistic	gl	Sig.
dif	0.166	22	0.118	0.895	22	0.023
difhum	0.175	22	0.079	0.879	22	0.011

^a^. Lilliefors significance correction.

**Table 5:** Correlation table.

		TempM	TempD
TempM	Pearson correlation	1	0.602
	Sig. (bilateral)		0.0018
	N	24	24
TempD	Pearson correlation	0.602	1
	Sig. (bilateral)	0.0018	
	N	24	24

Where TempM corresponds to the manual temperature and TempD corresponds to the temperature taken by the device.
